# Multifocal community-acquired MRSA infection: a rare case involving urinary tract, bloodstream, chest wall, and pleura

**DOI:** 10.1097/MS9.0000000000004161

**Published:** 2025-10-22

**Authors:** Jiansong Zhang, Chang Liu, Huaiyu Tian, Yi Ren

**Affiliations:** Department of Thoracic Surgery, Shenyang Chest Hospital, Shenyang, China

**Keywords:** antimicrobial resistance genes, chest wall abscess, community-acquired MRSA, multifocal infection, *Staphylococcus aureus* infections

## Abstract

**Introduction:**

Methicillin-resistant *Staphylococcus aureus* (MRSA) is a common cause of hospital- and community-acquired multidrug-resistant infections. Typical infection sites include the skin, respiratory tract, and bloodstream. This report describes a rare case of multifocal community-acquired MRSA (CA-MRSA) infection simultaneously causing urinary tract infection, bacteremia, chest wall abscess, and empyema.

**Case presentation:**

A 51-year-old man with newly diagnosed diabetes mellitus on admission was admitted with a 2-week history of right posterior chest wall pain and a mass. Imaging showed soft tissue swelling in the right chest wall and pleural effusion. Urine and blood cultures grew MRSA. The patient was treated with linezolid and levofloxacin, insulin therapy, and nutritional support, and the chest wall abscess was incised and drained. Pus culture and targeted next-generation sequencing (tNGS) confirmed MRSA infection with *mecA* and *SCCmec* antimicrobial-resistance genes. The patient recovered and had no recurrence during 2 months of follow up.

**Discussion:**

This case reveals that CA-MRSA can cause disseminated infections without obvious predisposing factors such as catheterization or trauma. Hyperglycemia and HHV-6 suggest impaired immune function. Combination antimicrobial therapy and surgical intervention were effective in the short term; however, the short follow-up period limits assessment of long-term outcomes. tNGS played a crucial role in identifying antimicrobial-resistance mechanisms.

**Conclusion:**

CA-MRSA can cause multifocal infections in immunocompromised patients. This rare case, with simultaneous urinary, bloodstream, chest wall, and pleural involvement, highlights the importance of early recognition, individualized therapy, and confirmation of resistance using tNGS. The outcome demonstrates the short-term effectiveness of this combined approach.

## Introduction

Methicillin-resistant *Staphylococcus aureus* (MRSA) is a multidrug-resistant Gram-positive pathogen, discovered in the 1960s, that is an important cause of both hospital- and community-acquired infections worldwide^[1]^. MRSA is highly pathogenic and transmissible, causing a range of infections such as bacteremia, pneumonia, and skin and osteoarticular infections, especially in immunocompromised individuals^[2]^. In recent years, the rise of community-acquired MRSA (CA-MRSA), which affects individuals without prior medical exposure, has led to the involvement of increasingly diverse infection sites^[3]^.

**HIGHLIGHTS**

- We report a case of multifocal community-acquired MRSA causing urinary tract infection, bacteremia, chest wall abscess, and empyema.
- The patient had no history of catheterization or urinary abnormalities, suggesting MRSA urinary colonization and hematogenous dissemination.
- Hyperglycemia and HHV-6 detection using targeted next-generation sequencing (tNGS) suggested underlying immunosuppression.
- The patient recovered after treatment with linezolid and levofloxacin and surgical drainage.
- tNGS confirmed the presence of *mecA* and *SCCmec* antimicrobial-resistance genes, demonstrating the utility of tNGS for surveillance of antimicrobial resistance.

MRSA-induced primary chest wall abscesses are rarely diagnosed owing to their subtle clinical signs and non-specific imaging findings, which can lead to missed diagnosis or misdiagnosis^[4,5]^. MRSA-induced urinary tract infections in individuals without catheterization or urinary abnormalities are rare. The presence of systemic infection or bacteremia, diabetes, immunosuppression, and antibiotic overuse are important contributing factors^[6]^.

To our knowledge, no cases of MRSA causing simultaneous urinary tract infection, bacteremia, primary chest wall abscess, and empyema have been reported to date. We report a case of MRSA infection at multiple sites in a man with previously undiagnosed poorly controlled diabetes and no other predisposing factors. The infection pathway and resistance mechanisms were identified through imaging, pathogen culture, and targeted next-generation sequencing (tNGS). This case highlights the potential for MRSA to cause multifocal invasive infections at atypical sites in immunocompromised patients. Recently, the TITAN 2025 guidelines have been proposed to ensure transparency, integrity, and accountability in the declaration and use of artificial intelligence in medical research and publishing^[7]^. This case report has been reported according to the CARE criteria^[8]^.

## Case report

A 51-year-old man was admitted with a 2-week history of a painful right posterior chest wall mass. The patient had no prior diagnosis of diabetes or urinary tract disease, and no notable history of trauma or surgery. He had smoked 20 cigarettes per day for 30 years. Laboratory testing on admission revealed severe hyperglycemia and a glycated hemoglobin (HbA1c) of 10.0%, leading to a new diagnosis diabetes mellitus.

Two weeks prior to admission, the patient had developed unexplained persistent dull pain in the right posterior chest, which became progressively worse and was accompanied by swelling and fever (highest temperature 38.6°C). He was treated with piperacillin-tazobactam (2.0 g, twice daily for 7 days) at a local hospital, but his symptoms did not improve, and he was referred to our hospital for chest magnetic resonance imaging, which suggested a chest wall tumor or tuberculosis. Three days prior to admission, he had developed increased pain and swelling but no cough, shortness of breath, or urinary symptoms.

**Figure 1.**
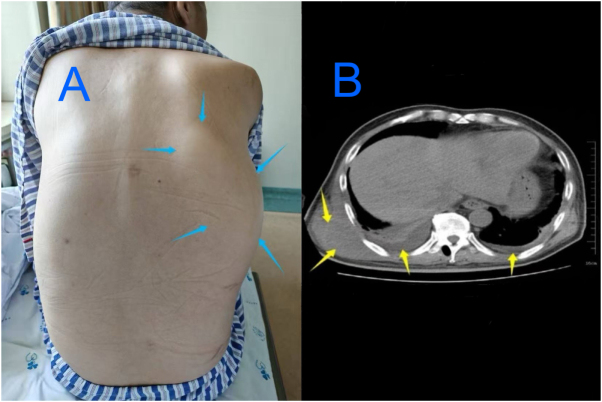
Images of the chest wall abscess and pleural effusion. (A) Photograph of the right chest wall abscess. The blue arrows indicate the margins. (B) Chest computed tomography scan showing a soft tissue mass in the right chest wall and bilateral small pleural effusions, more marked on the right side (yellow arrows).

Physical examination revealed firm swelling of the right posterior chest wall (approximately 15 cm in diameter), with ill-defined boundaries (Fig. 1a). The swelling was tender with elevated skin temperature, but no redness or fluctuation. His vital signs were stable, with a heart rate of 83 bpm.

Laboratory test results showed an elevated white blood cell count (14.16 × 10^9^/L), and C-reactive protein (206.92 mg/L), procalcitonin (3.3 ng/mL), hemoglobin (90 g/L), fasting blood glucose (23.30 mmol/L), glycated hemoglobin (HbA1c, 10.02%), and albumin (26.2 g/L) levels, and a urinary white blood cell count of 1237/μL.

Chest computed tomography showed soft tissue swelling of the right chest wall and mild bilateral pleural effusion (more marked on the right side) (Fig. 1b). Chest ultrasound revealed a mixed-echo area with a sinus tract (approximately 0.6 cm) connecting to a fluid collection in the pleural cavity. The urinary system ultrasound revealed no abnormalities.

On admission, systemic infection was suspected, and treatment was initiated with ticarcillin-clavulanate, short-acting insulin, and albumin. The abscess was not drained immediately owing to the patient’s hyperglycemia and poor nutritional status. Midstream urine and blood samples both tested positive for MRSA after 3 days of culture. Antibiotic sensitivity testing showed resistance to penicillin, nafcillin, clindamycin, and erythromycin, and susceptibility to linezolid, levofloxacin, gentamicin, and vancomycin. Based on the culture and sensitivity results, the antibiotic regimen was switched to oral linezolid 600 mg daily combined with levofloxacin 0.2 g twice daily. After the patient’s blood glucose levels and nutritional status improved, the chest wall abscess was incised and drained. An incision was made along the intercostal space, slightly below the center of the mass at the point of maximal fluctuation. After layer-by-layer dissection, a large amount of yellowish-white pus was drained from the abscess cavity, and fibrous septa and necrotic tissue were removed. No communication was found between the abscess and pleural cavity. Povidone-iodine irrigation was performed. The cavity was packed with gauze for drainage, which was changed every second day postoperatively.

**Figure 2.**
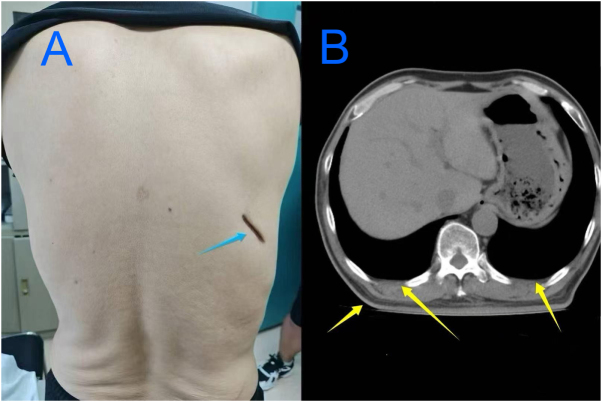
Follow-up findings 2 months after incision and drainage of the chest wall abscess. (A) Photograph of the right posterior chest wall showing the healed incision site (blue arrow). (B) Chest computed tomography scan showing resolution of the chest wall abscess and a reduction in the size of the bilateral pleural effusions (yellow arrows).

Pus culture and tNGS confirmed MRSA infection with *mecA* and *SCCmec* antimicrobial-resistance genes, and human herpesvirus 6 (HHV-6) infection. On postoperative day 7, the patient’s white blood cell count (7.13 × 10^9^/L), and body temperature normalized. He was discharged on postoperative day 10 and continued oral antibiotics for a further 4 weeks. By the 2-month follow-up the surgical wound had healed with no signs of infection (Fig. 2). The patient was offered the opportunity to share his personal experience of diagnosis and treatment in accordance with the CARE guidelines but declined.

## Discussion

MRSA infections manifest primarily as skin and soft tissue infections, pneumonia, osteomyelitis, and bacteremia^[9]^. This case demonstrates a rare instance of CA-MRSA causing multi-site infection, including urinary tract infection, bacteremia, chest wall abscess, and empyema. To our knowledge, this is the first report of CA-MRSA causing multi-site infection of these sites. This case expands knowledge of the clinical spectrum of MRSA infections and suggests that MRSA may exhibit greater invasiveness and dissemination potential in individuals with uncontrolled diabetes.

The patient had no history of urinary catheterization and no structural abnormalities of the urinary tract, suggesting that the infection might have originated from urinary tract colonization, and spread hematogenously to the chest wall and pleural cavity. Hyperglycemia promotes the development and spread of MRSA infection by inhibiting immune cell function and enhancing biofilm formation^[10]^. The concurrent detection of HHV-6, a virus that impairs immunity by infecting T and NK cells^[11]^, suggests that the patient was immunocompromised. This might have contributed to his susceptibility to invasive infection.

Chest wall and pleural infections caused by MRSA are rarely reported and difficult to diagnose. A few case reports of empyema necessitans due to MRSA complicated by metastatic spread such as osteomyelitis have demonstrated the pathogen’s invasive potential^[12]^. Similarly, a case of MRSA-positive sternoclavicular septic arthritis mimicking mediastinal malignancy, with diagnosis confirmed only after biopsy and culture, highlights the importance of considering MRSA in atypical thoracic infections^[13]^. A report of multiple MRSA-related cold abscesses of the chest wall with rib destruction, demonstrates the capacity of MRSA to cause severe destructive lesions in immunocompromised hosts^[14]^. This report demonstrates the critical role of early recognition, molecular diagnostics, and tailored therapy in managing multifocal CA-MRSA infections.

Although vancomycin is traditionally considered the first-line therapy for MRSA infection^[15]^, its tissue penetration and biofilm penetration are limited^[16]^. Linezolid, with broad distribution and high oral bioavailability, is more effective for treating MRSA infections and is better tolerated^[17]^. In this case, treatment with linezolid and levofloxacin resulted in rapid improvement of symptoms and inflammatory markers, demonstrating the effectiveness of this regimen. Surgical intervention was also crucial for treating the chest wall abscess. The pus, urine, and blood cultures were all positive for MRSA, suggesting that a single strain was disseminated to multiple sites. tNGS detected the *mecA* and *SCCmec* antimicrobial-resistance genes, highlighting the importance of molecular diagnostics in guiding treatment.

This case study has some limitations. The diagnosis was delayed owing to delayed abscess drainage. The short follow-up period of 2 months does not allow the long-term outcomes such as recurrence or chronic sequelae to be assessed. This limitation should be considered when interpreting the effectiveness of treatment in this case. Nevertheless, the lack of recurrence during the 2-month follow-up indicates good short-term control.

## Conclusion

This case report extends the spectrum of anatomical sites known to be affected by MRSA infection. To our knowledge, this is the first report of CA-MRSA simultaneously involving the urinary tract, bloodstream, chest wall, and pleura. Early diagnosis, individualized antimicrobial therapy, blood glucose control, surgical drainage, and molecular confirmation by tNGS were key to successful management, and demonstrates the effectiveness of this combined approach. Strengthened surveillance and infection control efforts both within and outside healthcare settings are needed to limit the spread and increasing virulence of MRSA.

## Data Availability

Not applicable.
